# SHP2 inhibition enhances Yes-associated protein–mediated liver regeneration in murine partial hepatectomy models

**DOI:** 10.1172/jci.insight.159930

**Published:** 2022-08-08

**Authors:** Ryan D. Watkins, EeeLN H. Buckarma, Jennifer L. Tomlinson, Chantal E. McCabe, Jennifer A. Yonkus, Nathan W. Werneburg, Rachel L. Bayer, Patrick P. Starlinger, Keith D. Robertson, Chen Wang, Gregory J. Gores, Rory L. Smoot

**Affiliations:** 1 Department of Surgery,; 2 Division of Biomedical Statistics and Informatics, and; 3 Division of Gastroenterology and Hepatology, Mayo Clinic, Rochester, Minnesota, USA.; 4 Department of Surgery, Medical University of Vienna, Vienna, Austria.; 5 Division of Molecular Pharmacology and Experimental Therapeutics and; 6 Department of Biochemistry and Molecular Biology, Mayo Clinic, Rochester, Minnesota, USA.

**Keywords:** Hepatology, Therapeutics, Drug therapy, Phosphoprotein phosphatases, Surgery

## Abstract

Disrupted liver regeneration following hepatectomy represents an “undruggable” clinical challenge associated with poor patient outcomes. Yes-associated protein (YAP), a transcriptional coactivator that is repressed by the Hippo pathway, is instrumental in liver regeneration. We have previously described an alternative, Hippo-independent mechanism of YAP activation mediated by downregulation of protein tyrosine phosphatase nonreceptor type 11 (PTPN11, also known as SHP2) inhibition. Herein, we examined the effects of YAP activation with a selective SHP1/SHP2 inhibitor, NSC-87877, on liver regeneration in murine partial hepatectomy models. In our studies, NSC-87877 led to accelerated hepatocyte proliferation, improved liver regeneration, and decreased markers of injury following partial hepatectomy. The effects of NSC-87877 were lost in mice with hepatocyte-specific *Yap/Taz* deletion, and this demonstrated dependence on these molecules for the enhanced regenerative response. Furthermore, administration of NSC-87877 to murine models of nonalcoholic steatohepatitis was associated with improved survival and decreased markers of injury after hepatectomy. Evaluation of transcriptomic changes in the context of NSC-87877 administration revealed reduction in fibrotic signaling and augmentation of cell cycle signaling. Cytoprotective changes included downregulation of *Nr4a1*, an apoptosis inducer. Collectively, the data suggest that SHP2 inhibition induces a pro-proliferative and cytoprotective enhancement of liver regeneration dependent on YAP.

## Graphical abstract

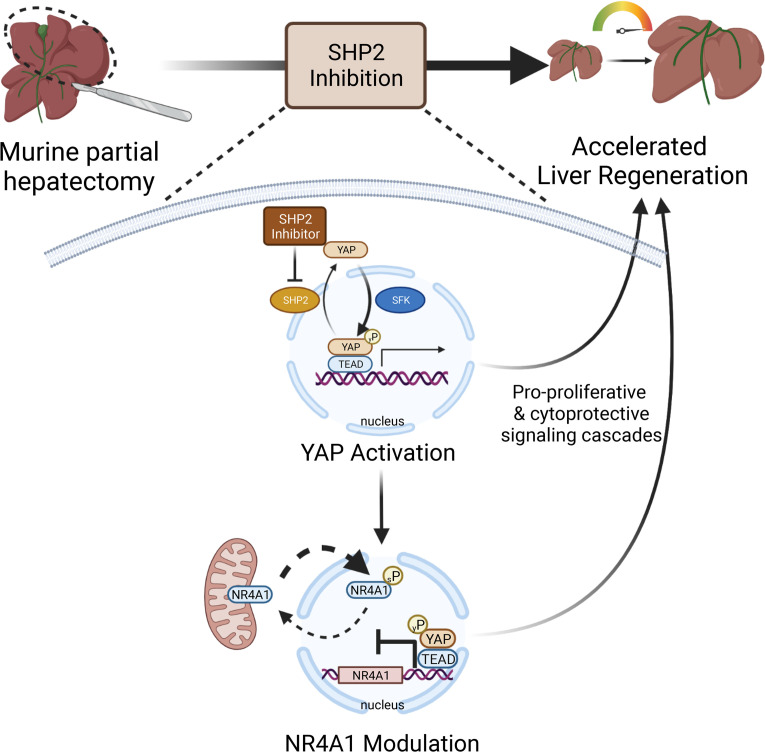

## Introduction

The liver’s intrinsic capacity to regenerate is a highly complex and tightly regulated process that enables recovery from various insults (1). This specialized capability is leveraged routinely in the setting of surgical resection, performed for both malignant and benign conditions (2). A minimum volume of functional liver remnant is required for regeneration. Determination of this threshold is a complex and difficult task based on patient-specific characteristics, such as baseline liver function (3). Underestimation of the required remnant liver can have significant consequences, such as hepatic insufficiency and acute hepatic failure known as posthepatectomy liver failure (PHLF) (3–5). PHLF has high rates of mortality, and there are no effective treatment options aside from liver transplantation, for which most patients do not qualify (6, 7). Therefore, adequate liver regeneration is a major determinant of a patient’s recovery following resection.

Nonalcoholic fatty liver disease (NAFLD) represents a spectrum of disease associated with obesity and altered metabolic signaling that ranges from simple steatosis to nonalcoholic steatohepatitis (NASH) (8). NASH is among the risk factors associated with PHLF (7). This risk factor is especially relevant as the prevalence of NAFLD is increasing worldwide, and in the United States, it is currently estimated that 30% of the population has NAFLD (9). Consistent with the clinical outcomes of patients with NAFLD and NASH, preclinical work has demonstrated that partial hepatectomy in murine models of NAFLD and NASH is associated with markedly increased mortality and impaired liver regeneration (10). Notably, there are no approved pharmacologic options to improve liver regeneration whether NAFLD is present or not.

While individual signaling events associated with liver regeneration are well studied, the mechanisms underlying coordination of this complex process are not fully understood (11–13). Therapeutic targeting of various pathways has been undertaken in murine partial hepatectomy models utilizing either genetic or small molecule approaches (14–19). A target of interest in regenerative medicine is Yes-associated protein (YAP), a transcriptional coactivator and regulator of organ size (20). The role of YAP in liver regeneration is well described (1, 21, 22). Canonically, YAP is regulated by the inhibitory Hippo pathway. Deletion or inhibition of the Hippo pathway in the liver leads to YAP activation, increased cellular proliferation, and enhanced liver regeneration (1, 18, 23). We have previously described an alternative pathway in which YAP is activated by tyrosine phosphorylation on tyrosine 357 (Y357) via the Src family kinase LCK (24–26). This activation mechanism can be opposed by tyrosine-protein phosphatase nonreceptor type 11 (SHP2). SHP2 inactivates YAP by dephosphorylating the Y357 residue, which promotes nuclear export and decreased transcriptional coactivity (24). Consequently, SHP2 inhibition is associated with increased YAP cotranscriptional activity and proliferation (24, 27). To our knowledge, activating YAP through SHP2 inhibition has not been explored previously in the context of liver regeneration.

Herein, we investigated the effects of SHP2 inhibition, utilizing the small molecule inhibitor NSC-87877 (NSC), on liver regenerative capacity in murine models of partial hepatectomy. These studies suggest that SHP2 inhibition is associated with a proregenerative, cytoprotective signaling program that results in augmented liver regeneration and improved survival.

## Results

### NSC accelerates liver regeneration following partial hepatectomy.

**Figure 1:**
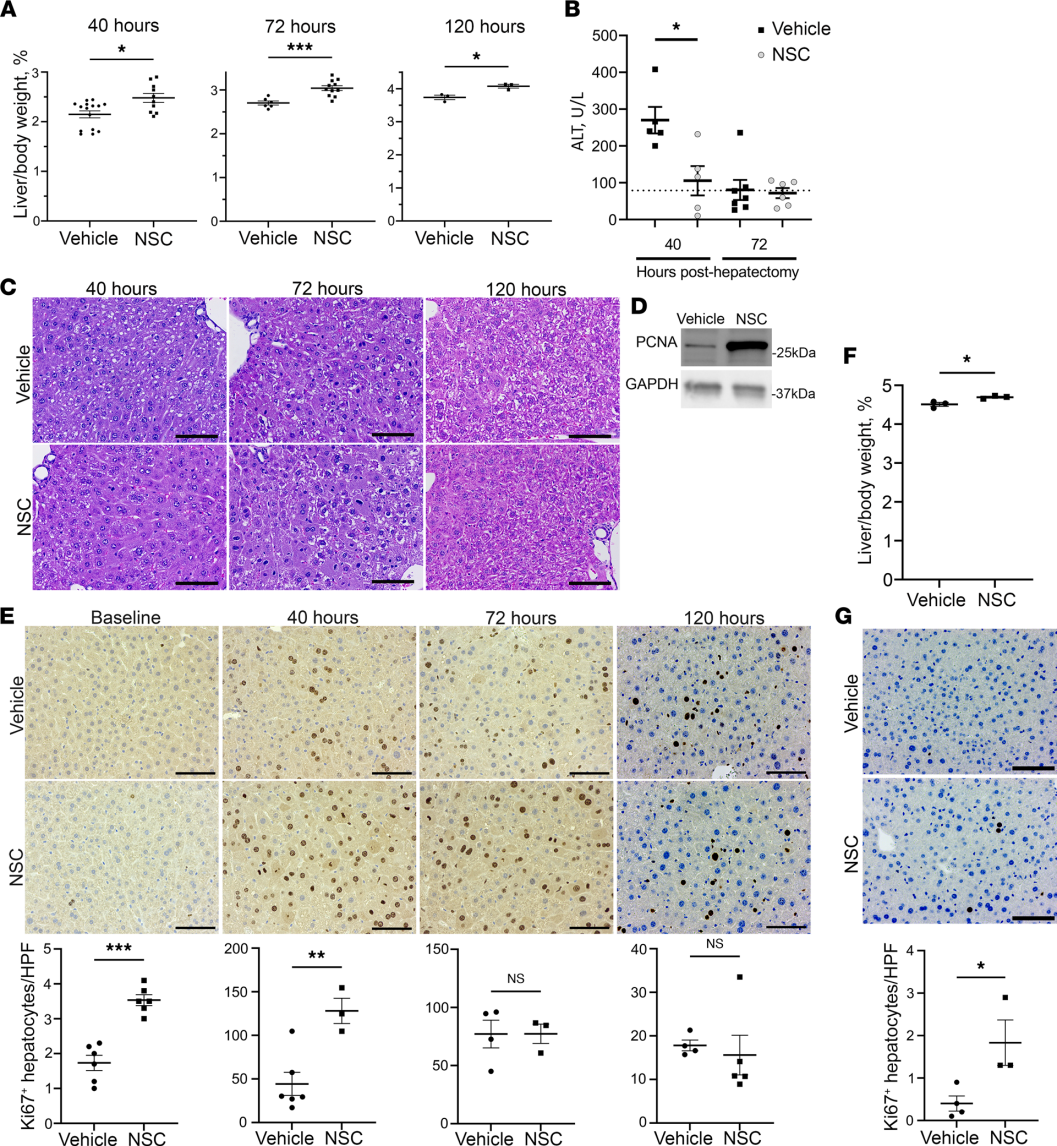
NSC-87877 accelerates liver regeneration following murine partial hepatectomy. (**A**) Mean liver/body weight ratio in mice treated with vehicle or NSC (7.5 mg/kg/dose, twice daily) 40 hours (*n* = 10-15), 72 hours (*n* = 6-11), and 120 hours (*n* = 3) after hepatectomy. (**B**) Plasma alanine aminotransferase (ALT) (U/L) 40 and 72 hours after hepatectomy (*n* = 5-7) in mice treated with vehicle or NSC. Dotted line represents the upper limit of normal (79 U/L). (**C**) Representative H&E-stained liver sections 40, 72, and 120 hours after hepatectomy in vehicle- and NSC-treated mice. Scale bar: 100 μm. (**D**) Proliferating cellular nuclear antigen (PCNA) immunoblot from murine whole liver lysates, 40 hours after hepatectomy, with GAPDH as a loading control. Representative of image of 2 independent experiments. (**E**) Representative Ki67 IHC-stained liver sections in vehicle- and NSC-treated mice from baseline, 40, 72, and 120 hours after hepatectomy (*n* = 3) quantified in 10 HPF (200×) per section. Scale bar: 100 μm. (**F**) Mean liver/body weight ratio in vehicle or NSC-treated mice following 4 weeks of treatment (*n* = 3). (**G**) Representative Ki67 IHC-stained liver sections in vehicle- and NSC-treated mice after 4 weeks of treatment. Ki67^+^ hepatocytes were quantified in 10 HPF (200×) (*n* = 3). Data are shown as mean ± SEM (*P < 0.05, **P < 0.01, ***P < 0.001). Statistical analysis was performed with 2-tailed Student *t* test.

Utilizing a standard 70% partial hepatectomy model in C57BL/6J mice (Supplemental Figure 1A; supplemental material available online with this article; https://doi.org/10.1172/jci.insight.159930DS1), we observed that NSC significantly increased the liver/body weight ratio at 40, 72, and 120 hours after hepatectomy, suggesting accelerated liver regeneration (Figure 1A). The uniformity of hepatectomy between groups was confirmed by assessing the mass of the resected liver in each mouse (Supplemental Figure 1B). Plasma was collected after hepatectomy, liver, and kidney function assessments were performed by measurement of alanine aminotransferase (ALT), alkaline phosphatase, total bilirubin, and blood urea nitrogen (BUN). Notably, early ALT levels were 2.5-fold higher in vehicle-treated mice compared with NSC-treated mice (Figure 1B), suggesting reduced hepatocyte injury in the NSC-treated mice. Alkaline phosphatase, total bilirubin, and BUN were not significantly different between groups and were within the normal range (Supplemental Figure 1C). Expectedly, mice lost weight in the first 3 postoperative days, and this was similar between treatment groups (Supplemental Figure 1D). We also evaluated the histomorphology of the regenerating liver to assess for structural changes associated with the accelerated regeneration. No gross histological changes in the liver architecture were observed (Figure 1C). Thus, NSC administration accelerated liver regeneration and reduced ALT levels after hepatectomy.

Given the increased liver/body weight ratio in NSC-treated mice, we next examined indices of hepatocyte proliferation. Proliferative markers were assessed in livers 40 hours after hepatectomy, which has been previously defined as a point in which there is peak hepatocyte mitotic activity (28). At this time point, we observed increased proliferating cellular nuclear antigen (PCNA) by immunoblot analysis in whole liver lysates and an increased number of Ki67^+^ hepatocytes, identified histologically (Figure 1, D and E). These data suggest that NSC can increase the number of hepatocytes that reenter the cell cycle at a time point associated with peak mitotic activity in a regenerating liver. Additionally, increased Ki67 staining was observed in the resection specimen, 12 hours after the first dose of NSC, which suggested that NSC may induce hepatocyte proliferation in the absence of hepatectomy (Figure 1E). To further explore this possibility, we administered NSC (7.5 mg/kg/dose, twice daily) or vehicle for 4 weeks to C57BL/6J mice and then evaluated liver/body weight ratio and Ki67 staining. We observed an increase in both liver/body weight ratio and Ki67^+^ cells in mice treated with NSC, further supporting that SHP2 inhibition induces proliferation in the absence of proregenerative stimuli (Figure 1, F and G). Extended treatment with NSC did not result in any significant changes in mouse bodyweight (Supplemental Figure 1E). There was also no change in gross or microscopic liver histomorphology; there was no evidence of neoplasia or increase in hepatic fibrosis (Supplemental Figure 1, F and G). These data support a model in which NSC appears to be well tolerated perioperatively and to be associated with accelerated liver regeneration in vivo.

### SHP2 inhibition activates YAP and mediates accelerated regeneration.

**Figure 2:**
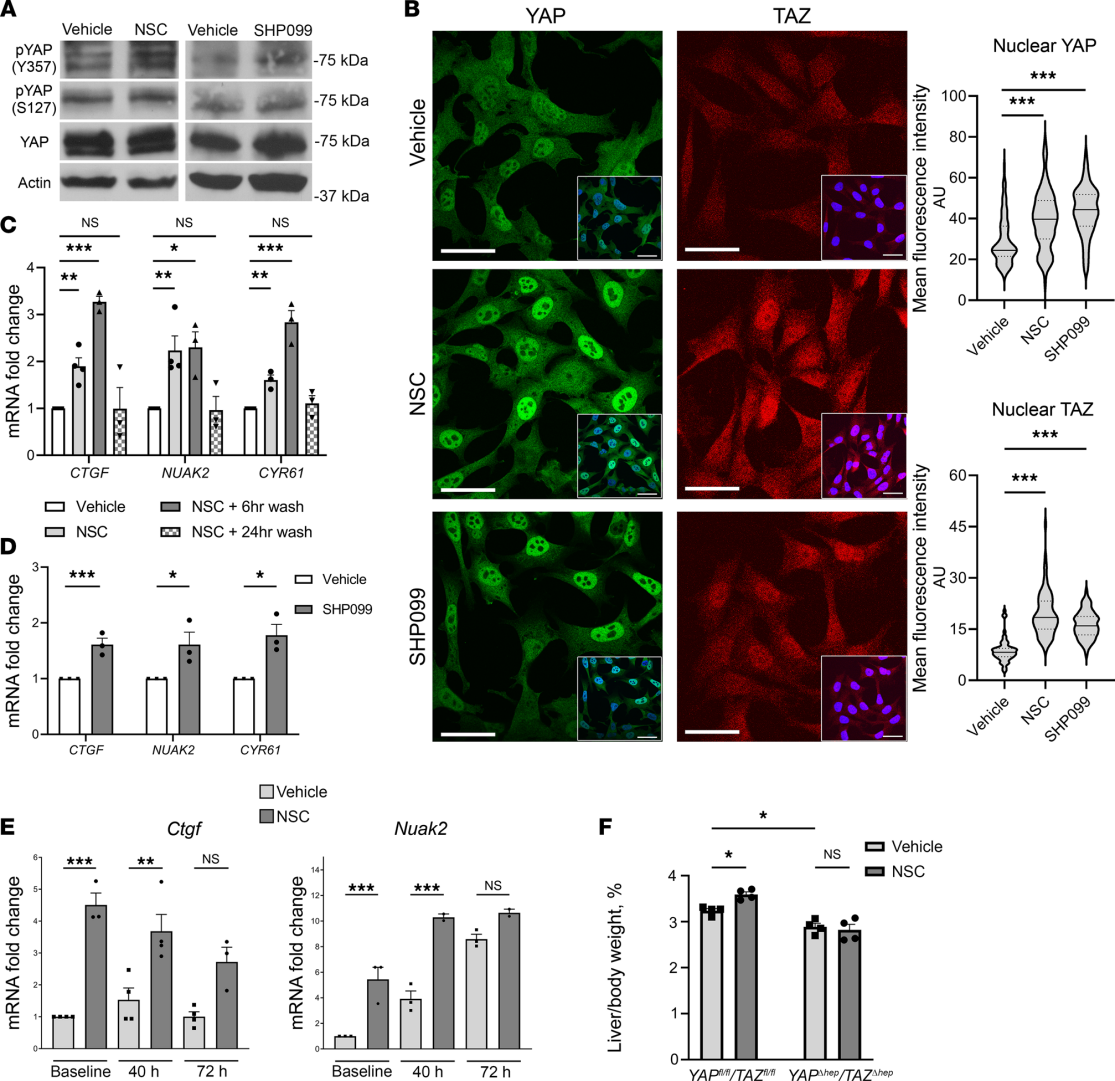
SHP2 inhibition activates YAP/TAZ in vitro and in vivo. (**A**) Hu1545 whole cell lysates treated with vehicle, NSC (1 μM for 24 hours), or SHP099 (1 μM for 24 hours) probed for pYAP^Y357^, pYAP^S127^, total YAP, and actin as a loading control. (**B**) Representative image of YAP and TAZ immunofluorescence stains in Hu1545 treated with vehicle, NSC (1 μM), or SHP099 (1 μM). Inset image with DAPI overlay. Scale bar: 50 μm for both inset and main image. YAP and TAZ mean fluorescence intensity (MFI) from 75 to 100 nuclei/condition plotted as a violin plot (solid line, median; dashed line, upper or lower quartile). (**C**) Hu1545 YAP/TAZ target gene expression (*CTGF*, *NUAK2*, and *CYR61*) following treatment with vehicle, NSC (1 μM for 24 hours), and NSC with 6- or 24-hour washout (*n* = 3–4). Data are shown as mean ± SEM. (**D**) Hu1545 YAP/TAZ target gene expression in vehicle- or SHP099-treated cells (1 μM for 24 hours) (*n* = 3). (**E**) YAP/TAZ target genes *Ctgf* and *Nuak2* from livers treated with vehicle or NSC at baseline (resection specimen), 40, and 72 hours after hepatectomy (*n* = 3). (**F**) Mean liver/body weight ratio 72 hours after hepatectomy in *Yap*^fl/fl^/Taz^fl/fl^ and *Yap^Δhep^*/Taz*^Δhep^* mice treated with vehicle or NSC (*n* = 4). Data are shown as mean ± SEM unless otherwise specified (*P < 0.05, **P < 0.01, ***P < 0.001). Statistical analysis was performed with 1-way ANOVA (**B**, **C**, **E**, and **F**) and 2-tailed Student *t* test (**C**).

Our prior studies in cholangiocarcinoma have identified that YAP activation and cellular proliferation is enhanced by SHP2 inhibition (24). Thus, we explored whether SHP2 inhibition altered YAP signaling, specifically in a noncancerous paradigm. We first assessed YAP signaling in Hu1545, a TERT-immortalized human hepatocyte cell line (29). In this cell line, we observed increased YAP tyrosine phosphorylation (pYAP^Y357^), a known activating posttranslational modification, in NSC-treated cells (Figure 2A). Notably, there was no significant change in serine phosphorylated YAP (pYAP^S127^), the Hippo pathway–specific phosphorylation that negatively regulates YAP activity. Increased pYAP^Y357^ is known to promote nuclear retention. Accordingly, we observed enrichment of YAP in the nucleus following NSC treatment in Hu1545 cells (Figure 2B). The posttranslational modifications of WW domain–containing transcription regulator 1 (TAZ) — a YAP paralog — that were not delivered by the Hippo pathway are not sufficiently studied. However, we observed concurrent nuclear enrichment of TAZ in Hu1545 cells following treatment with NSC or SHP099 (Figure 2B). This nuclear enrichment resulted in increased YAP/TAZ-dependent cotranscriptional activation, as evidenced by increased expression of YAP/TAZ target genes *CTGF*, *NUAK2*, and *CYR61* (Figure 2C). To understand the reversibility of YAP/TAZ activation, expression of YAP/TAZ target genes were assessed in NSC-stimulated Hu1545 cells following a subsequent washout period. NSC-stimulated cells maintained or further increased YAP/TAZ target gene expression 6 hours after removal of NSC. Cognate gene expression returned to baseline 24 hours after removal of NSC, indicating that the activation of YAP/TAZ was reversible (Figure 2C).

We exposed an additional transformed normal liver cell line, normal human cholangiocytes (NHC), to NSC and evaluated YAP activation, where we similarly observed increased abundance of pYAP^Y357^ and YAP cotranscriptional activity (Supplemental Figure 2, A and B). Prior work has demonstrated that SHP2 subcellular localization can change with YAP/TAZ modulation due to complex formation between these proteins (30). Interestingly, we did not observe any alteration in subcellular localization of SHP2 after NSC or SHP099 treatment in Hu1545 cells (Supplemental Figure 2C).

NSC also inhibits SHP1 (31). Inhibition of SHP1 has been identified as an activator of Src family kinases that results in YAP activation (32). To address this possibility, we examined the effects of a structurally dissimilar selective SHP2 inhibitor, SHP099 (33), on the cellular activation of YAP in Hu1545 cells. Following exposure to SHP099, we observed similar effects on YAP phosphorylation, YAP/TAZ localization, and cognate gene expression as observed after NSC administration (Figure 2, A, B, and D). Additionally, we evaluated the effects of NSC and SHP099 on Src activity directly by immunoblot in Hu1545 cells. We observed decreased Src phosphorylation (Src^Y416^), an activating posttranslational modification, following treatment with NSC and SHP099 (Supplemental Figure 3A). These findings suggest that YAP activation is a unique result of SHP2 inhibition.

SHP2 has been demonstrated to be involved in mediating RAS/MAPK pathway activation. The phosphatase activity and scaffolding function of SHP2 have both been implicated in this process (34). Previous studies have demonstrated that NSC affects different downstream effectors of RAS/MAPK signaling. Specifically, p38 signaling was downregulated in neuroblastoma via inhibition of dual-specificity protein phosphatase 26 (DUSP26) (35), mitogen induced ERK1/2 activation was downregulated in breast cancer cells (31), and JNK activity was downregulated in CD8^+^ T cells in murine encephalomyelitis (36). We evaluated these signaling cascades in Hu1545 cells following treatment with NSC or SHP099. The JNK and p38 signaling pathways did not significantly change following treatment with NSC or SHP099 (Supplemental Figure 3B). We did observe increased ERK activity with NSC treatment and diminished activity with SHP099 (Supplemental Figure 3B). These results indicate that NSC activity only modifies ERK1/2 activation in vitro and no other downstream MAPK pathway effectors. The effects on ERK1/2 are likely a result of SHP1 inhibition, based on previous studies in SHP1 ablated hepatocytes (37).

To assess how SHP2 inhibition affected YAP activity in vivo, we examined YAP/TAZ target gene expression in the liver resection specimen and regenerating remnant at 40 and 72 hours after hepatectomy. We observed increased expression of *Ctgf* and *Nuak2* in the resection specimen and the regenerating remnant 40 hours after hepatectomy (Figure 2D). Target gene transcripts were not significantly different between vehicle- and NSC-treated mice 72 hours after hepatectomy but were increased from baseline (Figure 2E). In vitro, YAP/TAZ activation was reversible, but we sought to understand if YAP/TAZ activity returned to baseline after the normal murine regenerative course. Mice were randomized to perioperative treatment with NSC or vehicle and underwent hepatectomy, followed by 7 days of twice-daily treatment. Posthepatectomy days 8–14, mice did not receive any additional treatments, and this is labeled as the washout period. Mice were then euthanized on day 14 after hepatectomy (Supplemental Figure 4A). Liver/body weight ratios were not statistically different between groups (Supplemental Figure 4B). YAP/TAZ target genes *Ctgf* and *Nuak2* levels were not statistically different between treatment groups, nor were they different from baseline expression levels (Supplemental Figure 4C). Histological analysis did not reveal any gross neoplasia, structural differences, or fibrosis in NSC-treated mice (Supplemental Figure 4D). These results indicate that YAP activation occurs early in the regenerative timeline and is transient.

We further evaluated if YAP activation was a major contributing mechanism by which accelerated liver regeneration occurred following NSC administration. To explore this, we performed hepatocyte-specific deletion of YAP and its paralog TAZ concurrently, utilizing *Yap^fl/fl^*/*Taz^fl/fl^* mice injected i.v. with hepatocyte tropic AAV8 viral particles expressing Cre recombinase (38). These *Yap*^Δhep^/*Taz*^Δhep^ mice then underwent treatment with NSC or vehicle perioperatively (Supplemental Figure 4E). YAP/TAZ knockdown efficiency was confirmed by immunoblot of whole liver lysates (Supplemental Figure 4F). *Yap^fl/fl^*/*Taz^fl/fl^* mice were utilized as an additional control for the specific genetic background. Notably, liver regeneration was accelerated by NSC in the *Yap^fl/fl^*/*Taz^fl/fl^* mice, similar to WT mice (Figure 2F). In contrast, NSC did not induce changes in posthepatectomy liver mass in *Yap*^Δhep^/*Taz*^Δhep^ mice compared with vehicle (Figure 2F). These data support that SHP2 inhibition activates YAP both in vitro and in vivo and that YAP/TAZ are critical signaling components downstream of SHP2 inhibition that induce accelerated liver regeneration.

### SHP2 inhibition induces a proregenerative transcriptional profile.

**Figure 3:**
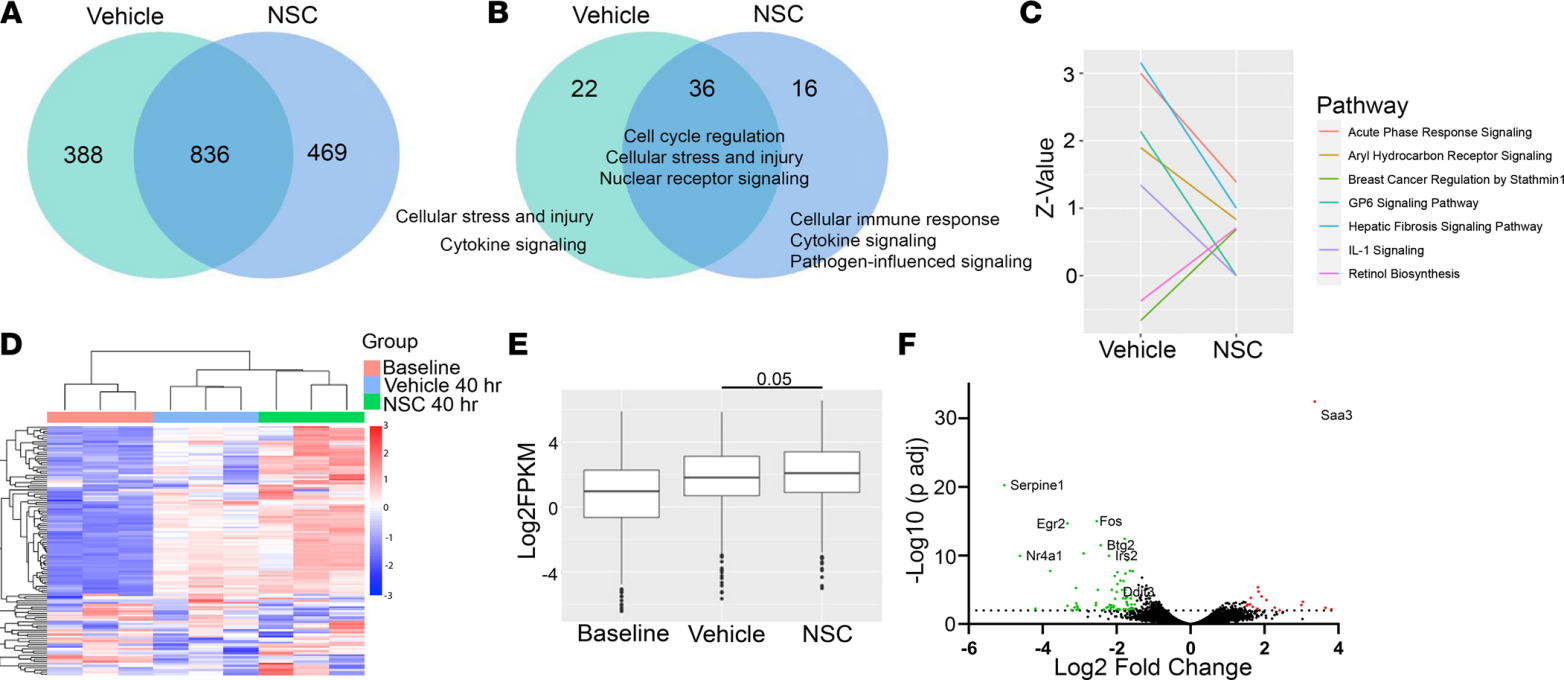
SHP2 inhibition induces a proregenerative transcriptional profile. (**A**) Venn diagram of differentially regulated genes (–1.5 < log_2_ fold change > 1.5, FDR < 5%) in murine liver lysates comparing baseline to 40 hours after hepatectomy in vehicle- and NSC-treated mice (*n* = 3). (**B**) Common function of IPA canonical pathways significantly changed (–log_10_ *P* > 1.3) 40 hours after hepatectomy from baseline in vehicle- and NSC-treated mice. The most common functions identified are listed. The full pathway analysis is provided in Supplemental Table 1. (**C**) IPA canonical pathway activation scores (*Z* score) in pathways in which the pathway was significantly changed from baseline (–log_10_ *P* > 1.3), and the *Z* score changed by greater or less than 1 and –1, respectively. (**D**) Cell cycle gene set heatmap at baseline (vehicle resection specimen) and 40 hours after hepatectomy in mice treated with vehicle or NSC. Each column represents an individual biological replicate. (**E**) Box and whisker plot (median and IQR) log_2_FPKM of cell cycle gene transcripts from heatmap in **E**. (**F**) Volcano plot comparing gene expression 40 hours after hepatectomy in vehicle- and NSC-treated mice (3 biological replicates/group). Significantly differentially expressed genes (–1.5 < log_2_FC > 1.5, FDR < 5%) highlighted green (downregulated) and red (upregulated) with top genes identified on plot. Statistical analysis was performed using Wilcoxon rank sum test with continuity correction (**E**).

YAP appears to be central to the acceleration of liver regeneration observed with SHP2 inhibition; however, we sought to further define downstream signaling changes induced by NSC treatment in an unbiased manner through bulk RNA-Seq. Transcriptional changes were evaluated by RNA-Seq of whole liver samples (resection specimen and remnant) at 40 hours after hepatectomy in vehicle- and NSC-treated WT mice. The total number of genes differentially expressed 40 hours after hepatectomy in vehicle- and NSC-treated mice was similar (1224 versus 1305), with a similar percentage of genes upregulated in vehicle- and NSC-treated mice (66.2% versus 64.2%). However, a unique subset of genes was differentially regulated in NSC-treated mice (Figure 3A). Ingenuity pathway analysis (IPA) was utilized to evaluate the function of the 3 groups of differentially regulated genes: genes shared between groups, genes unique to vehicle-treated mice, and genes unique to NSC-treated mice. The functions, based on canonical pathway analysis, of the shared genes was enriched in pathways involved in cellular proliferation and cell cycle control. The functions of the unique NSC canonical pathways included cytokine signaling and cellular immune response (Figure 3B); all IPA canonical pathways are listed in Supplemental Table 1. To evaluate changes in specific IPA canonical pathway activation between the genes unique to vehicle- and NSC-treated mice, we plotted the change in activation profile (IPA canonical pathway *Z* score). Comparisons were made between groups in which the pathway was significantly modified in one of the groups (Figure 3C). Notably, hepatic fibrosis pathway signaling activation was decreased in the NSC-treated mice. Genes that mapped to this pathway included *Timp1*, *Tgfb2*, *Serpine1*, and *Fos*. Consistent with these observations, genetic ablation of TGF-β or TIMP1 have previously been shown to improve liver regeneration after hepatectomy (39, 40). Reduction in IL-1 signaling and increased stathmin signaling was also observed. IL-1 signaling has been shown to inhibit cellular proliferation after hepatectomy (41); stathmin signaling has been associated with proliferating hepatocytes (42). We utilized this strategy to evaluate MAPK signaling pathways in vivo. The p38 MAPK signaling was enriched in NSC-treated mice when compared with baseline (*P* = 0.02) and trended toward increased activity in vehicle-treated mice (*P* = 0.07). However, the difference in activation scores was only 0.41. Pathway activation in ERK/MAPK signaling and SAPK/JNK were not significantly altered in either group. These data indicate that NSC induces a proregenerative transcriptome in pathways not traditionally associated with YAP and does not significantly alter MAPK signaling pathways in vivo.

Due to the increased Ki67 IHC in WT mice, we hypothesized that NSC treatment augmented the expression of cell cycle genes normally induced after hepatectomy. Cell cycle genes were identified based on a standard cell cycle gene set (KEGG Cell Cycle, 04110). The majority of gene transcripts in this set were upregulated in regenerating livers after hepatectomy, and these pro-proliferative transcripts were further induced in NSC-treated mice (Figure 3, D and E). Overall, this transcriptomic profiling revealed that NSC treatment induced an enhanced proliferative state after hepatectomy, while altering pathways in fibrosis and inflammatory signaling.

### SHP2 inhibition modulates NR4A1 and is cytoprotective.

Orphan nuclear receptor subfamily 4 group A member (NR4A1), alias Nur77, is involved in a broad spectrum of cellular processes, such as apoptosis and cellular proliferation (43). *Nr4a1* is of specific interest, as its transcription has been shown to be negatively correlated with YAP activity (44). In these prior studies, increased YAP activity using genetic manipulation of the Hippo pathway modulated NR4A1. Active YAP also induced phosphorylation of NR4A1, indirectly through AKT, which directed NR4A1 away from mitochondria and to the nucleus. This resulted in reduced apoptosis and increased proliferation (44). The role NR4A1 plays in liver regeneration has only been explored in models of genetic manipulation, in which NR4A1 KO improved regeneration (44, 45).

**Figure 4:**
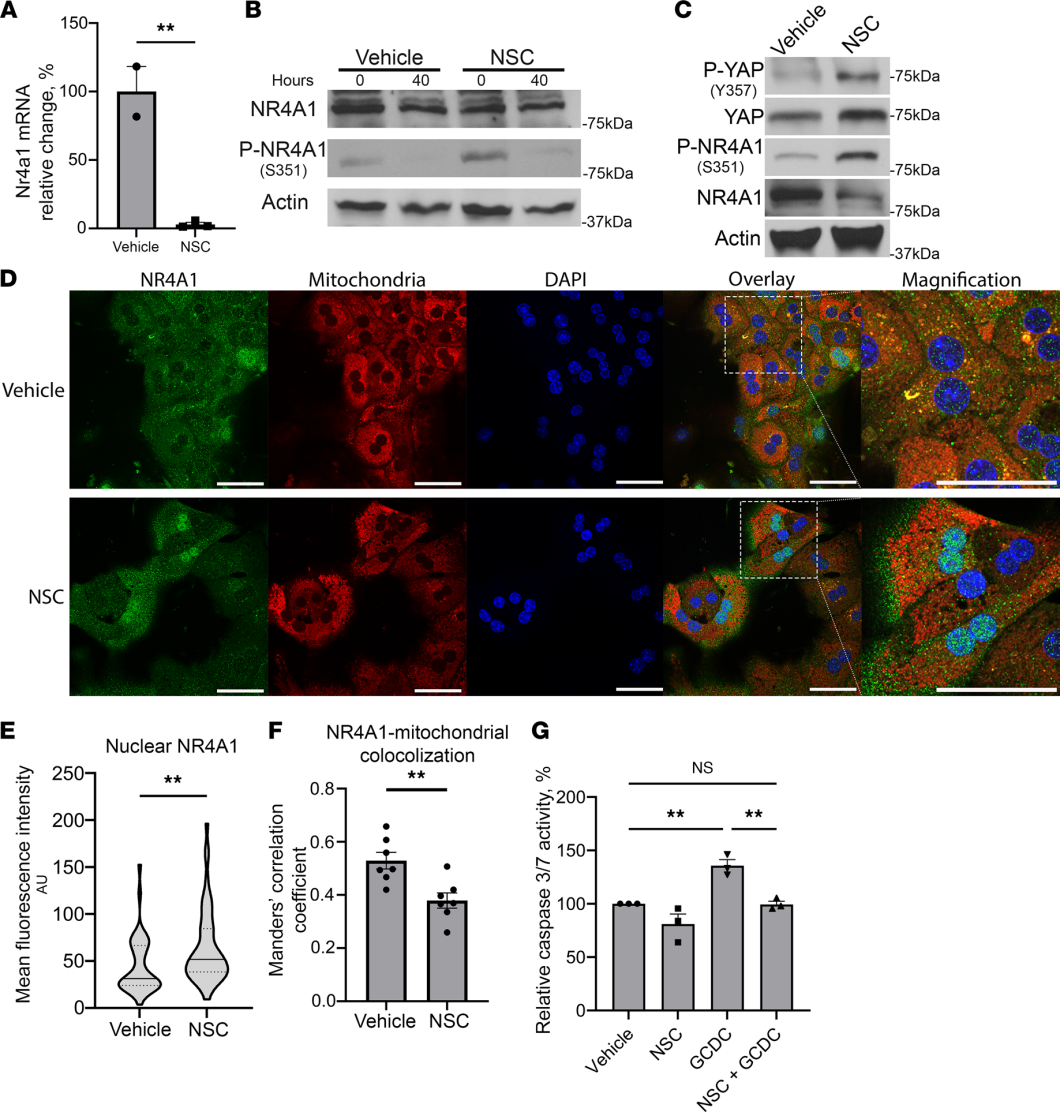
NR4A1 in modulated by NSC in vitro and in vivo. (**A**) *Nr4a1* mRNA relative expression 40 hours after hepatectomy in vehicle- and NSC-treated mice (*n* = 3). Data are shown as mean ± SEM. (**B**) Liver lysates from resection specimen (0 hours) and 40 hours after hepatectomy probed for NR4A1, pNR4A1^S351^, and actin as a loading control in vehicle- and NSC-treated mice. (**C**) Primary mouse hepatocytes treated with NSC (10 μM) or vehicle for 24 hours and immunoblotted for pYAP^Y357^, total YAP, pNR4A1^S351^, total NR4A1, and actin as a loading control. (**D**) Representative images of NR4A1 immunocytochemistry with mitochondrial (MitoTracker) and nuclei (DAPI) counterstains in isolated mouse hepatocytes treated with NSC (10 μM) or vehicle. Scale bars: 50 μm. (**E**) Mean fluorescence intensity (MFI) values for nuclear NR4A1 in NSC- (10 μM) or vehicle-treated hepatocytes. Median values represented by solid line, and IQR represented by dotted lines. In total, 35–50 nuclei were evaluated. (**F**) Mean Manders’ correlation coefficient of NR4A1 colocalized to mitochondria in vehicle- and NSC-treated hepatocytes. Correlation coefficient were calculated for 7 images (400×). Data are shown as mean ± SEM. (**G**) Relative caspase 3/7 activity in primary mouse hepatocytes 4 hours after glycochenodeoxycholate (GCDC) (50 μM) or vehicle treatment with or without NSC pretreatment (10 μM) for 16 hours, *n* = 3 (*P < 0.05, **P < 0.01). **B** and **C** are representative immunoblots from 2 independent experiments. Statistical analysis was performed with 2-tailed Student *t* test (**E** and **F**) and 1-way ANOVA (**G**).

We observed *Nr4a1* as one of the top downregulated genes between vehicle- and NSC-treated mice, 40 hours after hepatectomy (Figure 3F). This finding was confirmed by quantitative PCR (qPCR) (Figure 4A). In WT mouse liver lysates, NSC increased the serine phosphorylation of NR4A1 (pNR4A1^S351^) in the resection specimen (0 hours) (Figure 4B). These transcriptional and posttranslational changes indicate increased YAP activity, but whether this modulated NR4A1 localization was unknown. To explore if the NR4A1 phosphorylation changes observed in vivo induce subcellular redistribution, we stimulated isolated primary mouse hepatocytes in vitro with NSC. YAP activation and NR4A1 modulation was observed as indicated by increased pYAP^Y357^, increased pNR4A1^S351^, and reduction in total NR4A1 (Figure 4C). Confocal microscopy confirmed that these changes resulted in NR4A1 redistribution from mitochondria to the nuclei of hepatocytes following NSC exposure (Figure 4, D–F).

The reduced ALT levels observed in vivo suggested that NSC induced a cytoprotective effect. To evaluate this, we evaluated the apoptotic response of isolated primary hepatocytes to a toxic bile acid stimulus with or without NSC pretreatment. Hepatocytes exposed to a toxic dose of glycochenodeoxycholate (GCDC) (50μM) expectedly had increased caspase 3/7 activity, indicative of apoptosis. However, cells pretreated with NSC did not exhibit increased caspase 3/7 activity (Figure 4G). These data indicate that NSC may protect hepatocytes from toxic apoptotic stimuli by modulating NR4A1 signaling; this cytoprotection may aid liver regeneration induced by the surgical process.

### SHP2 inhibition improves postoperative mortality in mice with NASH.

**Figure 5:**
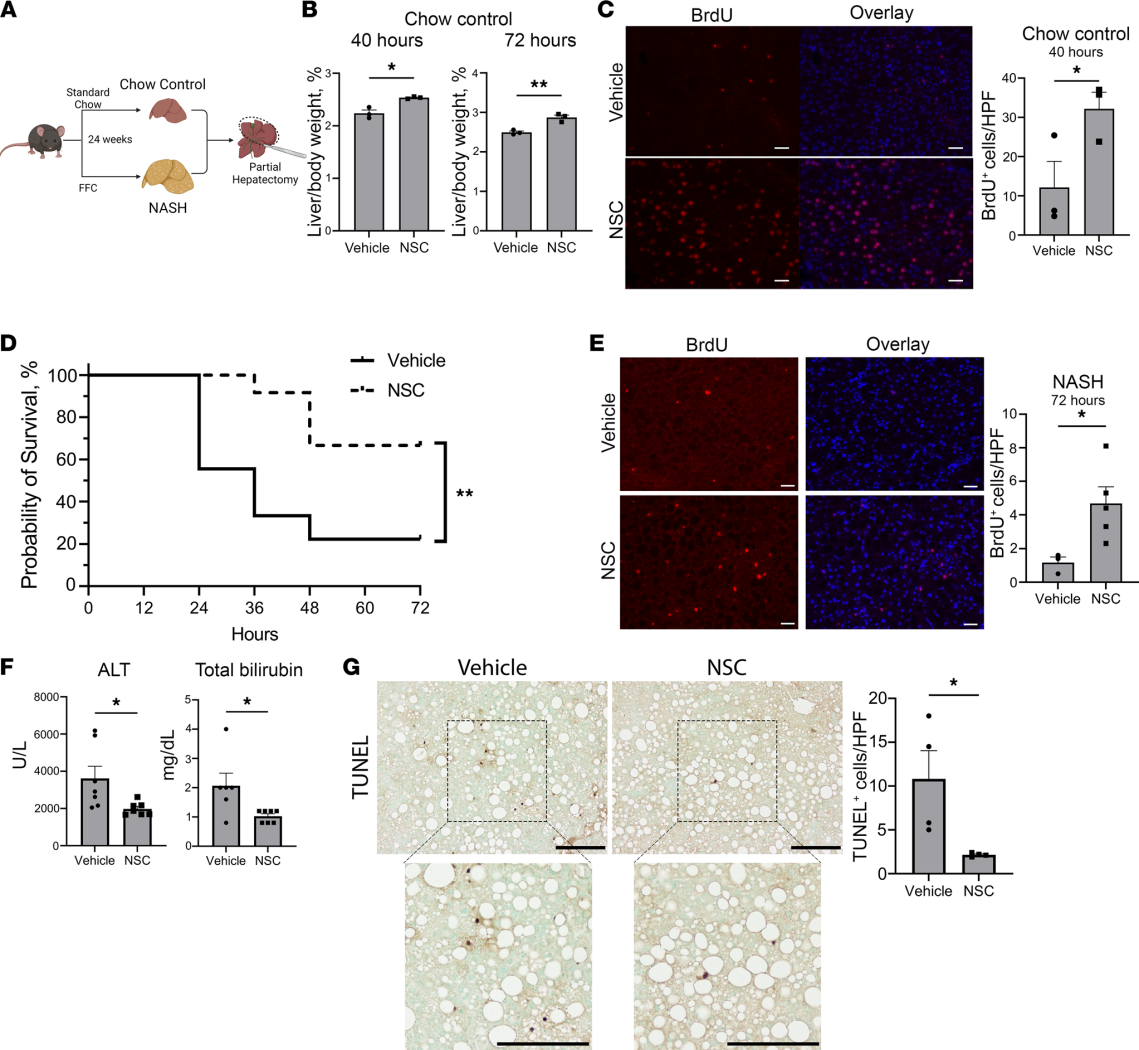
NSC improves postoperative mortality in murine NASH model. (**A**) NASH induction with a high-fat, -cholesterol, -glucose/sucrose diet (FFC). (**B**) Mean chow control mouse liver/body weight ratio 40 and 72 hours after hepatectomy (*n* = 3). (**C**) Representative BrdU immunofluorescence staining in liver sections 40 hours after hepatectomy in vehicle- and NSC-treated chow control mice (*n* = 3). Scale bar: 100 μm. DAPI-counterstained cells overlayed with BrdU with quantified BrdU^+^ hepatocyte nuclei per 10 HPF (200×). (**D**) Kaplan-Meier survival curve after hepatectomy in mice with NASH in vehicle- (*n* = 18) or NSC-treated (*n* = 12) mice. Statistical analysis was performed using the log rank (Mantel-Cox) test. (**E**) Representative BrdU immunofluorescence staining in liver sections 72 hours after hepatectomy in vehicle- and NSC-treated NASH mice (*n* = 3). Scale bar: 100 μm. DAPI-counterstained cells overlayed with BrdU. Quantified BrdU^+^ hepatocyte nuclei per 10 HPF (200×). (**F**) Plasma ALT and total bilirubin in NASH mice treated with vehicle or NSC 4 hours after hepatectomy (*n* = 6–7). (**G**) Representative image of TUNEL IHC in vehicle or NSC-treated NASH mice 4 hours after hepatectomy with magnified view. Scale bars: 200 μm. TUNEL^+^ hepatocytes were quantified in 10 HPF (100×) for each mouse (*n* = 4). Data are shown as mean ± SEM (*P < 0.05, **P < 0.01). Statistical analysis was performed with 2-tailed Student *t* test, unless otherwise specified.

Patients with hepatic steatosis have increased postoperative morbidity, while patients with steatohepatitis have higher morbidity and mortality after hepatectomy (46, 47). Due to this increased risk in patients with steatohepatitis, we explored the role of SHP2 inhibition in preventing postoperative mortality in mice with NASH. Mice were randomized to standard chow (chow controls) or a high-fat, -fructose, and -cholesterol (FFC) diet (Figure 5A) that induces NASH in mice and mimics the human NASH phenotype (48, 49). Phenotypic characterization of mice after 24 weeks of FFC diet demonstrated increased weight gain and gross liver size (Supplemental Figure 5, A and B). Concurrent, steatosis with ballooning hepatocytes and fibrosis was also observed (Supplemental Figure 5, C and D).

Despite their increased age, chow control mice treated with NSC had similar increased liver/body weight ratios after hepatectomy (Figure 5B). Proliferation was also enhanced as identified by increased BrdU incorporation in liver sections collected 40 hours after hepatectomy (Figure 5C). Notably, 70% partial hepatectomy in other models of murine NASH has led to poor posthepatectomy survival (10). In our model, NASH mice that underwent partial hepatectomy also had a poor survival rate of 22.2% 72 hours after hepatectomy. This survival rate is improved to 66.7% (*P* = 0.002) when NASH mice were administered NSC perioperatively (Figure 5D). NSC treatment in NASH mice did not result in increased liver mass 72 hours after hepatectomy. This is likely confounded by only evaluating mice that survived to the study endpoint and the heterogeneity in starting liver mass indicated by increased variability in resection volumes in NASH mice (Supplemental Figure 5, E and F). Histologic analysis of vehicle- or NSC-treated mice did not reveal any gross changes (Supplemental Figure 5G). Additionally, plasma analysis (ALT, total bilirubin, alkaline phosphatase, and BUN) did not identify any significant changes at this time point except a nonsignificant decrease in plasma cholesterol (247 mg/dL versus 169.75 mg/dL, *P* = 0.19, *n* = 3–4/group). However, treatment with NSC resulted in increased BrdU incorporation 72 hours after hepatectomy compared with vehicle-treated NASH mice (Figure 5E).

In a separate cohort of mice with diet-induced NASH, plasma and liver remnants were evaluated 4 hours after hepatectomy. Similar to WT mice, we observed significant reduction in ALT levels in addition to total bilirubin in NASH mice. Additionally, TUNEL IHC was performed on the liver remnant and displayed decreased TUNEL^+^ hepatocytes, further illustrating the cytoprotective role of NSC treatment early after hepatectomy in NASH (Figure 5, F and G). These data indicate that NSC treatment can improve postoperative survival in a clinically relevant disease model potentially through pro-proliferative and cytoprotective signaling cascades.

## Discussion

This study delineates a proregenerative, cytoprotective signaling cascade in murine livers initiated by SHP2 inhibition. Specifically, the major findings of this study include that (a) pharmacologic inhibition of SHP2 activates YAP and accelerates liver regeneration following partial hepatectomy in mice; (b) SHP2 inhibition results in transcriptional changes associated with pro-proliferative and metabolic signaling pathways; and (c) SHP2 inhibition improves survival in a NASH partial hepatectomy model. These findings suggest that SHP2 inhibition may have clinical utility in promoting liver regeneration.

Improving the liver’s capacity to regenerate has proven difficult, despite the growing knowledge base surrounding this highly complex and regulated process. No clinically available therapeutic agents exist, and impaired/dysfunctional liver regeneration continues to carry a high degree of morbidity and mortality, especially in the posthepatectomy setting (6, 7). Preclinical studies have demonstrated a central role for YAP in regulating liver regeneration; however, the exact mechanisms both upstream and downstream of YAP remain unresolved and contradictory (17, 23, 50–52). For example, TGF-β has been suggested to be both required for YAP-mediated regenerative signaling and to be detrimental (39, 52). Excessive YAP signaling has been associated with liver steatosis and fibrosis in some preclinical models (53, 54), but in a carbon tetrachloride injury model, YAP activation through MST1/2 inhibition decreased fibrosis (23). Thus, there may be context-specific signaling, leading to differences in overall responses. Adding another layer of complexity to understanding YAP signaling in liver regeneration are the differential dependencies of cell types within the liver on YAP. For example, previous work has demonstrated that hepatocyte-specific YAP deletion impairs regeneration to a minor degree, whereas cholangiocyte-specific YAP deletion causes cholestasis and severe defects in liver regeneration, highlighting the central role of YAP/TAZ in cholangiocyte biology (50). Our experiments partially recapitulate these findings. We observed a deficit in, but not complete elimination of, liver regeneration following hepatocyte specific YAP/TAZ deletion. However, we also found that regeneration could not be augmented by SHP2 inhibition in these hepatocyte-specific deletion models. These findings suggest that YAP/TAZ signaling in hepatocytes is not necessary for adequate liver regeneration but may be an important determinant of overall intensity of the regenerative response, allowing these pathways to be augmented pharmacologically. Whether this is a cell-autonomous or nonautonomous regulation is an important area of future investigation, as increased YAP signaling within hepatocytes has been previously shown to induce changes in the vascular microenvironment through upregulation of HGF/c-MET signaling in liver (55).

Direct comparison of transcriptional profiles expressed in vehicle- and NSC-treated mice 40 hours after hepatectomy identified additional proregenerative targets, independent of known YAP signaling pathways, that NSC altered. For example, *Saa3* transcript expression was increased following NSC administration. *Saa3* is an acute phase reactant specific to mice, a pseudogene in humans, that has been shown to be associated with liver regeneration following partial hepatectomy and may be important for metabolic adaptation and cellular protection (56, 57). Notable genes that were downregulated by NSC treatment included *Egr2* and *Ddit3*. *Egr2* downregulation has been associated with a metabolic shift in a hepatocyte-derived cell line that improved glucose uptake and decreased altered lipid metabolism associated with insulin resistance. Our data imply that improved metabolic signaling may be a benefit of NSC administration, as well (58). Additionally, *Ddit3* (alias CHOP), when genetically ablated, has been shown to improve survival and hepatocyte proliferation following a toxic acetaminophen dose (59). Thus, NSC may alter signaling cascades involved in cell death, metabolic signaling, and acute phase protein response after hepatectomy that may play a role in the enhanced liver regeneration observed in vivo.

In spite of unresolved mechanistic questions, the benefit of augmenting YAP signaling on tissue regeneration in general and in the liver specifically has been demonstrated (21, 22). Activation has been achieved utilizing both genetic approaches and small molecule inhibitors (17, 18, 23). For example, inhibition of MST1/2 kinases, which negatively regulate YAP activity via serine phosphorylation, is associated with increased markers of YAP activity, cellular proliferation, and liver/body weight ratio following partial hepatectomy in mice (23). MST1/2 inhibitors have also been associated with decreased injury in acetaminophen overdose models, dextran sulfate sodium (DSS) colitis models, and CCl_4_ administration models, suggesting overlap between regeneration and cytoprotection, similar to our observations. Activating YAP, in a Hippo pathway–independent manner, was previously unexplored in the context of liver regeneration. Utilizing 2 small molecule inhibitors, SHP2 inhibition resulted in YAP activation, and this activation in hepatocytes was central to the pro-proliferative and cytoprotective effects we observed. Notably, YAP and TAZ have diverse roles in various cell-specific contexts that include mechanosensing, cytoskeletal rearrangement, and immune cell trafficking (50, 60). Thus, the specific mechanisms contributing to the enhanced regeneration we observed will need to be evaluated in future studies; however, these preclinical data indicate that activating YAP through SHP2 inhibition represents a therapeutic strategy to accelerate liver regeneration.

Pharmacological inhibition of SHP2 resulting in YAP activation had not been explored previously. However, the role of SHP2 has been interrogated in murine liver regeneration. Bard-Chapeau et al. examined liver regeneration in mice with hepatocyte-specific SHP2 deletion (61). They observed decreased BrdU incorporation after hepatectomy; however, this did not result in significant differences in liver/body weight ratio. These results appeared to be mediated by reduced ERK1/2 signaling after hepatectomy. Our results differ from this previous study for several potential reasons. First, SHP2 scaffolding and enzymatic functions have both been implicated in downstream MAPK signaling. Genetic ablation permanently removes both of these functions, while pharmacological inhibition with NSC has been shown to only inhibit the enzymatic function of SHP2 (31). Next, we did not observe significant reduction of ERK1/2 signaling in our models, which was observed with their hepatocyte-specific deletion models. Finally, pharmacological inhibition of SHP2 with NSC is transient, as demonstrated in our washout studies, allowing for significant potential differences. Notably, Bard-Chapeau et al. (61) observed hepatoprotective effects of SHP2 ablation, similar to our observations.

YAP-directed therapy has theoretical risks, especially as it relates to neoplasia. Genetic downregulation of Hippo pathway components leads to liver overgrowth and, eventually, hepatocellular carcinoma (62–65). Additionally, activated YAP is oncogenic in murine models of cholangiocarcinoma (66). In fact, our initial exploration of SHP2 as a YAP regulatory molecule was conducted in cholangiocarcinoma models. We noted increased proliferation and resistance to chemotherapy following SHP2 inhibition/deletion, similar to findings where SHP2 deletion resulted in carcinogenesis in vivo (24, 67, 68). Given the known association between YAP activation and cancer, previous studies with MST1/2 inhibition included long-term administration without evidence of neoplasia (23). We noted similar findings in our studies with NSC. However, clinical indications requiring long-term administration would require additional safety studies. Clinically, an area of significant need is in the prevention and/or treatment of posthepatectomy liver insufficiency/failure. In this clinical situation, the expected duration of therapy would be short (typically a matter of days to weeks). Our results indicate that NSC induced YAP/TAZ activation transiently and returned YAP/TAZ activity to baseline both in vitro and in vivo. These results, paired with the need for short-term administration, likely limits the risk of neoplasia.

Overall, these results demonstrate that pharmacologic SHP2 inhibition is a feasible and tolerable option to accelerate liver regeneration and is cytoprotective. The findings of this study support this approach as a regenerative medicine therapeutic strategy.

## Methods

### Reagents.

NSC was purchased from Cayman Chemical (no. 56990-57-9). SHP099 was purchased from Selleck Chemicals (no. 1801747-42-1). GCDC was purchased from MilliporeSigma (no. G0759).

### Cell culture.

Hu1545 was provided by Keith Robertson (Mayo Clinic) and maintained in culture at 37°C and 5% CO_2_ in DMEM (Thermo Fisher Scientific) with 10% (v/v) FBS (Thermo Fisher Scientific), penicillin (100 IU/mL; Thermo Fisher Scientific), and streptomycin (100 μg/mL; Thermo Fisher Scientific). NHC were maintained in culture as previously described (69). These cells were provided by Nicholas LaRusso (Mayo Clinic). Experiments with NHC were carried out in DMEM/10% FBS. Primary mouse hepatocytes were isolated from mice by collagenase (Thermo Fisher Scientific) digestion followed by Percoll gradient purification (Thermo Fisher Scientific), as previously described, with minor modifications (70). Cells were plated on collagen-coated plates in DMEM with 10% FBS for 6 hours prior to experimental treatments.

### Animals.

C57BL/6J mice were obtained from the Jackson Laboratory for all experiments. Mice were maintained under a 12-hour light-dark cycle and fed ad libitum.

### NASH model.

Ten-week-old male C57BL/6J were housed 5 mice per cage, and cages were randomized to a diet for 24 weeks: standard chow (Diet Pico Laboratory Rodent Diet) or FFC diet, which included a high-fat and -cholesterol chow (AIN-76A Western Diet; 1810060; Test Diet)and drinking water that was supplemented with fructose (23.1 g/L, MilliporeSigma, F2543) and glucose (18.9 g/L,MilliporeSigma, 49158) as previously described (49).

### In vivo therapeutics.

For the murine partial hepatectomy model, mice were treated 1 day preoperatively and then every 12 hours postoperatively until euthanasia, unless otherwise noted (Supplemental Figure 1). Mice received NSC, 7.5 mg/kg every 12 hours (15 mg/kg/day) i.p., dissolved in normal saline. Control mice received equal volumes of normal saline.

### Partial hepatectomy model.

Male age-matched mice were randomly assigned to control or treatment. Surgical procedures were performed in small cohorts to ensure uniform timing in relation to the day/night cycle. Mice were anesthetized by vaporized isoflurane for an average operation time of 15 minutes. Two-thirds partial hepatectomy was conducted as previously described (71). Briefly, cholecystectomy followed by sequential ligation and excision of the left median, right median, and left lateral lobes was performed. Hemostasis was achieved. The abdomen was then closed in 2 layers with running 4-0 Vicryl. The resected tissue was collected for further molecular analysis as baseline. Liver regeneration was assessed 40, 72, and 120 hours after hepatectomy. All excised tissue was either frozen and stored at –80°C or fixed in 10% (v/v) buffered formalin overnight at room temperature.

### YAP/TAZ hepatocyte deletion in vivo.

*Yap/Taz–*double floxed mice (*Yap^fl/fl^*/*Taz^fl/fl^*) were obtained from The Jackson Laboratory (strain no. 030532). Eight-week-old *Yap^fl/fl^*/*Taz^fl/fl^* mice were administered 1 × 10^11^ AAV8 particles expressing AAV.TBG.PI.Cre.rBG, a gift from James M. Wilson (University of Pennsylvania, Philadelphia, Pennsylvania, USA; Addgene viral prep no. 100787-AAV8) as previously described, i.v. by tail vein injection (38). Four weeks following administration, mice underwent 70% partial hepatectomy as described above.

### Plasma analysis after hepatectomy.

Immediately following euthanasia, blood was collected via the inferior vena cava and placed in a lithium heparin tube. Plasma was separated from the blood by centrifugation at 2000*g* for 15 minutes at 4°C and transferred to new tubes. Plasma was aliquoted and stored at –80°C. Plasma was analyzed with Vetscan VS2 chemistry analyzer (Zoetis).

### RNA-Seq.

RNA was isolated from mouse livers using Qiagen RNeasy kit with on column DNase digestion per the manufacturer protocol. The raw RNA-Seq paired-end reads for the samples were processed through the Mayo RNA-Seq bioinformatics pipeline, MAP-RSeq version 3.1.4 (72). Briefly, MAP-RSeq employs the splice-aware aligner, STAR (73), to align reads to the reference human genome build hg38. The aligned reads were then processed through a variety of modules in a parallel fashion. Gene and exon expression quantification were performed using the Subread (74) package to obtain both raw and normalized (reads per kilobase per million mapped reads [RPKM]) reads. Finally, comprehensive analyses were run on the aligned reads to assess quality of the sequenced libraries. The data presented in this publication have been deposited to NCBI’s Gene Expression Omnibus (GEO) and are accessible through GEO series accession no. GSE198641. Using the raw gene counts report from MAP-RSeq, genes differentially expressed between the groups were assessed using the bioinformatics package edgeR 2.6.2 (75). Genes found different between the groups were reported, along with their magnitude of change (log_2_ scale) and their level of significance (FDR < 5%). Canonical pathway analysis was performed using IPA software (Ingenuity Systems). Biological functions and disease information within the IPA software were used to investigate the canonical pathways of interest.

### qPCR.

Total RNA was isolated from cultured cells or mouse liver samples using TRIzol, followed by isopropanol precipitation. Reverse transcription was performed with Moloney murine leukemia virus reverse transcriptase and random primers (Invitrogen). Real-time PCR (Light Cycler 480 II, Roche Diagnostics) was performed with Sybr Green (Roche Diagnostics), with primer sequences listed in Supplemental Table 3. Relative expression of target genes was calculated using the ΔΔCt method, with target gene normalization to the geometric mean of 18S rRNA expression.

### Western blot.

Proteins were isolated from mouse livers or cell lysates by mechanical disruption in cell lysis buffer (Cell Signaling Technology) with protease inhibitors (Roche), phosphatase inhibitors (Roche), and 1 mM PMSF (MilliporeSigma). Cellular debris was removed by centrifugation (12,000*g* for 15 minutes at 4°C). Proteins were resolved by SDS-PAGE on Tris-Glycine gels, followed by transfer to 0.2 μm nitrocellulose. Membranes were blocked in 5% BSA in TBS-Tween20 (0.1% v/v) and then stained overnight at 4°C. Primary antibodies are listed in Supplemental Table 2. Secondary HRP antibodies were applied for 1 hour at room temperature, and blots were visualized with ECL or ECL prime (GE Healthcare Life Sciences) chemiluminescence. Membranes were stripped, blocked, and reblotted as needed.

### Caspase 3/7 activity.

Primary mouse hepatocytes were plated (30,000 cells/well) and cultured in a black wall 96-well plate. Following treatment described in the corresponding figure legend (Figure 4G), caspase 3/7 activity was determined using Apo-ONE homogenous caspase-3/7 assay (Promega, G7792) according to manufacturer guidelines.

### Immunocytochemistry.

Cells were plated on coverslips and then treated. Cells were fixed in 4% (v/v) paraformaldehyde (PFA) for 30 minutes, followed by permeabilization with 0.1% (v/v) Triton X-100 in PBS for 5 minutes. Cells were blocked in 5% (w/v) BSA in PBS and incubated with primary antibody at 4°C overnight on a shaker. Primary antibodies listed in Supplemental Table 2. Secondary antibodies were then applied for 1 hour at room temperature, and nuclei were counterstained with DAPI. Coverslips were then mounted on glass microscope slides and then visualized by confocal microscopy (Zeiss LSM 780, Zeiss). Images utilized for mean fluorescence intensity calculations were obtained under identical conditions. In vitro, mitochondria were stained using MitoTracker Red CMXROS (M7512, Invitrogen). Briefly, cells were incubated with MitoTracker Red (400 nM) for 30 minutes and then fixed with 4% PFA at 37°C. Cells were then stained according to the protocol described above. Mean fluorescence intensity and colocalization was evaluated using ImageJ (NIH). Manders’ coefficient of correlation was calculated using the ImageJ plugin JACoP as previously described (76).

### BrdU labeling.

Two hours prior to euthanasia, mice were administered BrdU (100mg/kg) (MilliporeSigma) i.p. FFPE liver sections were incubated with primary antibody at 4°C overnight. Secondary antibodies were applied for 1.5 hours at room temperature, and nuclei were counterstained with DAPI. Ten high-powered fields (HPF; 40×) were visualized, and BrdU^+^ hepatocytes were counted on a immunofluorescence microscope (Invitrogen EVOS M5000).

### IHC.

Paraffin sections were cut at a thickness of 5 μm. Tissues were stained for histological analysis with H&E or primary antibodies listed in Supplemental Table 2. For quantification of Ki67^+^ hepatocytes, 10 HPF (20×) were visualized per sample and manually counted. TUNEL was performed according to the manufacturers protocol (Abcam, ab206386) in 10 μm FFPE liver sections. TUNEL^+^ cells were quantified in 10 fields of view (10×) for each section.

### Statistics.

Statistical analyses were performed with Prism version 9 (GraphPad Software) software or SPSS (IBM SPSS Statistics). Comparison of 2 groups for in vitro and in vivo studies was performed using 2-tailed Student *t* test. Comparison of 3 or more groups was performed using 1-way ANOVA with Bonferroni post hoc test. Survival analysis was performed using log rank (Mantel Cox) test in SPSS. *P* < 0.05 was considered statistically significant.

### Study approval.

All animal experiments were performed with Mayo Clinic IACUC approval.

## Author contributions

RDW contributed study concept and design; acquisition, analysis, and interpretation of data; and drafting of the manuscript. EHB and JLT contributed acquisition, analysis, and interpretation of data. CEM contributed analysis and interpretation of data, as well as technical support. JAY, NWW, and RLB contributed acquisition of data. PPS and CW contributed interpretation of data and critical revision of the manuscript for important intellectual content. KDR contributed material support and critical revision of the manuscript for important intellectual content. GJG contributed study concept and design, analysis and interpretation of data, critical revision of the manuscript for important intellectual content, material and technical support, and study supervision. RLS contributed study concept and design; analysis and interpretation of data; critical revision of the manuscript for important intellectual content; funding acquisition; administrative, material, and technical support; and study supervision.

## Supplementary Material

Supplemental data

Supplemental table 1

## Version 1 06/28/2022

In-Press Preview

## Version 2 08/08/2022

Electronic publication
