# Supplementary material for: SHP2 inhibition enhances Yes-associated protein–mediated liver regeneration in murine partial hepatectomy models
Source: JCI Insight. 2022 Aug 8;7(15):e159930. doi: 10.1172/jci.insight.159930 (PMC9462473; doi:10.1172/jci.insight.159930)
Supplement: Supplemental data [file jciinsight-7-159930-s103.pdf]

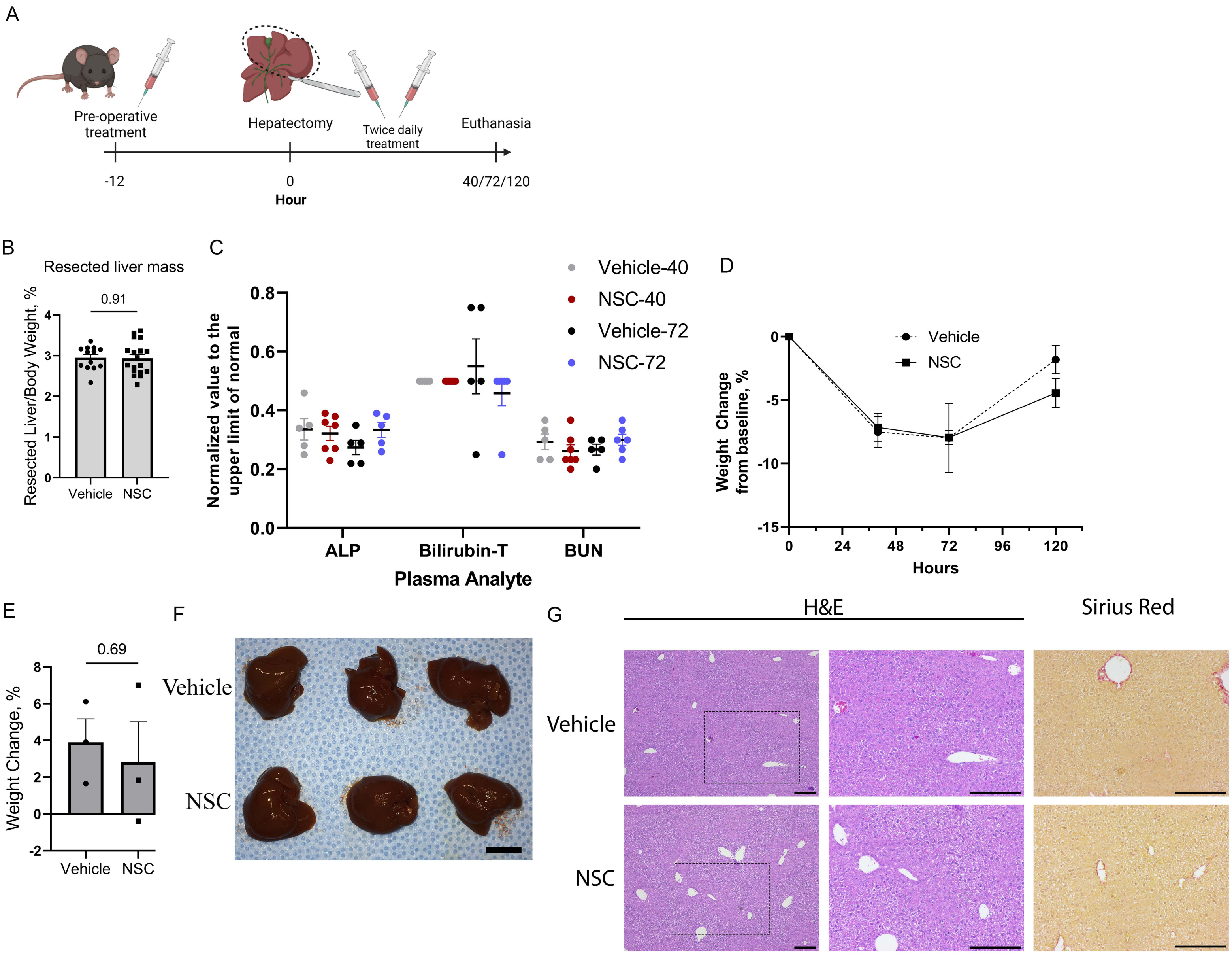

Supplemental Figure 1. NSC treatment pre- and post- partial hepatectomy is tolerable.

A) Treatment timing for partial hepatectomy models. **B)** Average resected liver mass normalized to body weight in vehicle and NSC treated wild-type mice (n=14-18). **C)** Plasma analysis (n=5-7) of alkaline phosphatase (ALP), total bilirubin (bilirubin-T), and blood urea nitrogen (BUN) in vehicle and NSC treated mice 40- and 72-hours post-hepatectomy. Plasma analytes normalized to upper limit of normal. **D)** Average weight change in mice 40- (n=10-15), 72- (n=6-11), and 120-hours (n=3) post-hepatectomy. **E)** Average weight change from baseline in mice treated with vehicle or NSC for 4 weeks (n=3). **F)** Photograph of mouse livers treated with vehicle or NSC for 4 weeks. Scale bar=1cm. **G)** Representative images of H&E at 40X and 100X magnification and Sirius Red stained liver section in mice treated with vehicle or NSC for 4 weeks (n=3). Scale bars=200µm. Graphs represent mean ± SEM. Statistical analysis was performed with student t test.

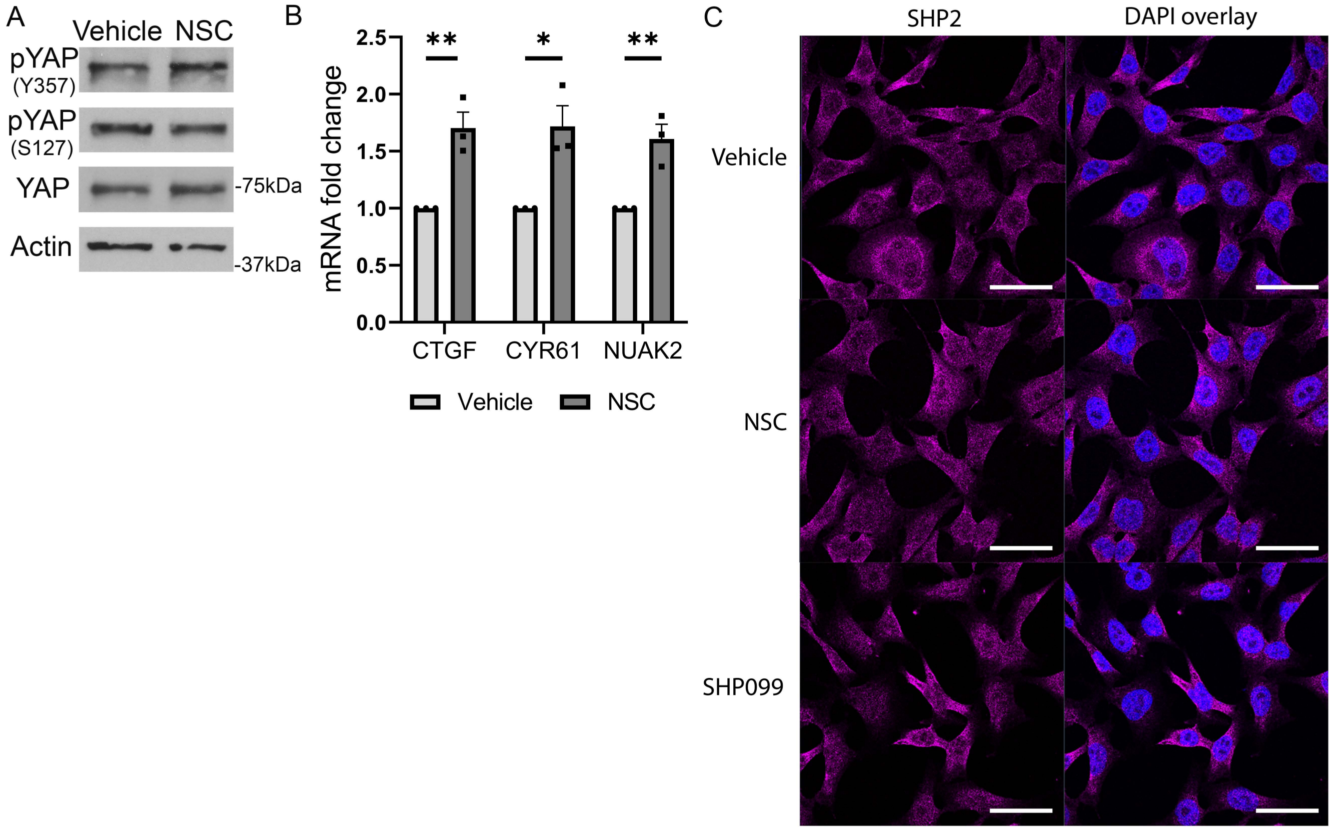

Supplemental Figure 2. SHP2 inhibition activates YAP in vitro without SHP2 subcellular modification

A) Normal human cholangiocyte (NHC) cell lysates treated with vehicle or NSC (1µM x 24 hours) immunoblot probed for $pYAP^{Y357}$, $pYAP^{S127}$, total YAP, and actin as a loading control. Representative immunoblot from one experiment. **B)** YAP target gene expression in NHC treated with vehicle or NSC (n=3). **C)** Hu1545 SHP2 immunocytochemistry with DAPI counterstain in cells treated with vehicle, NSC (1µM), or SHP099 (1µM). Scale bar =50µm. Graphs represent mean ± SEM (*p<0.05, **p<0.01). Statistical analysis was performed with student t test.

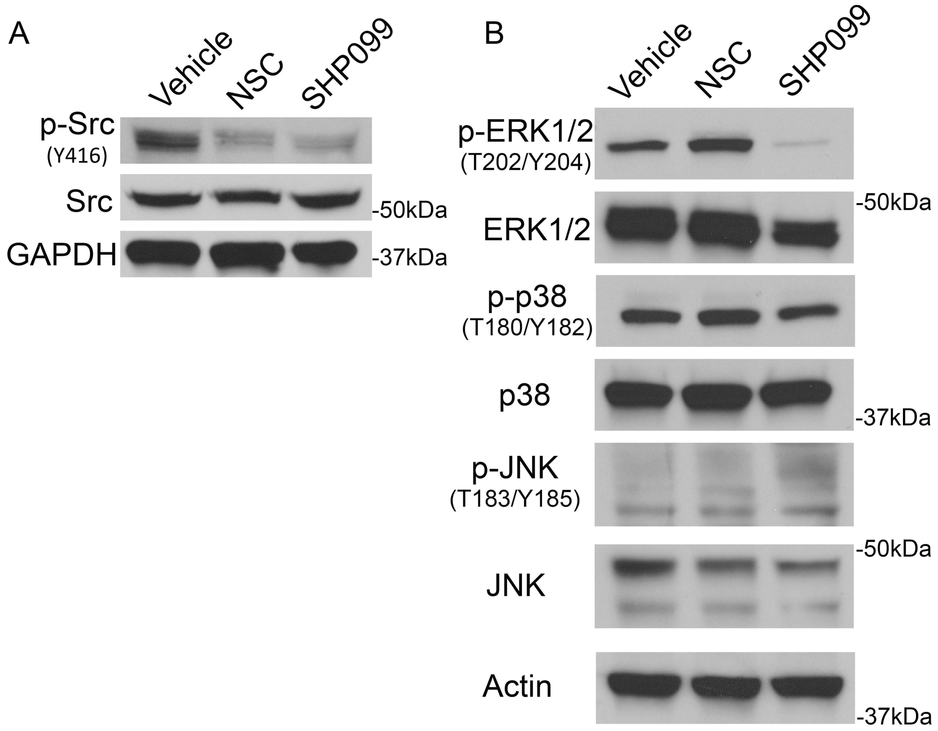

Supplemental Figure 3. SHP2 inhibition effects on Src and MAPK signaling pathway activation.

A) Hu1545 cell lysate immunoblot for p-Src following vehicle, NSC or SHP099 treatment (1µM x 24 hours) with Src and GAPDH as loading controls. **B)** Hu1545 cell lysate immunoblot for p-ERK1/2, p-p38, and p-JNK following vehicle, NSC or SHP099 treatment (1µM x 24 hours). Immunoblots are representative of 2 independent experiments.

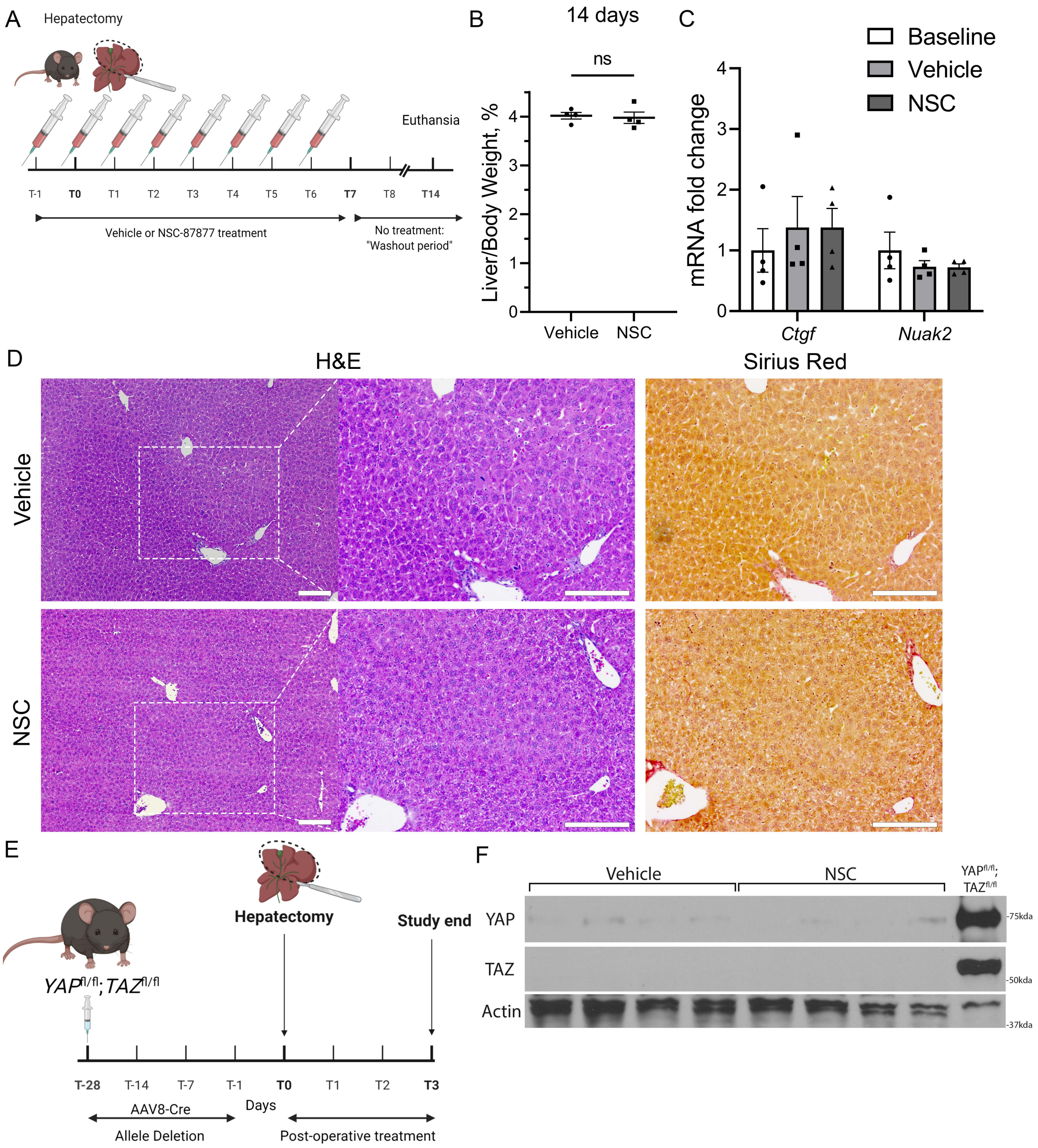

Supplemental Figure 4. NSC YAP/TAZ activation in vivo is reversible following partial hepatectomy.

A) Experimental schematic for in vivo NSC washout studies. **B)** Liver to body weight ratio 14-days post hepatectomy in mice treated with vehicle or NSC for 7 days post-hepatectomy. **C)** YAP/TAZ target genes (*Ctgf and Nuak2)* fold change from baseline in livers 14 days post-hepatectomy. **D)** Representative H&E at low and high power and Sirius red stained liver sections in mice 14 days post-hepatectomy. Scale bars=200µm. **E)** Schematic for the creation of *Yap*$^{\Delta hep}$/*Taz*$^{\Delta hep}$ by by AAV8-Cre intravenous injection. **F)** *Yap*$^{\Delta hep}$/*Taz*$^{\Delta hep}$ liver lysates probed for YAP and TAZ in mice treated with vehicle or NSC with *Yap*$^{fl/fl}$/*Taz*$^{fl/fl}$ as a control with actin as a loading control. Graphs represent mean ± SEM (*p<0.05, **p<0.01, ns=not significant (p>0.05)). Statistical analysis was performed with student t test (panel B) and one-way ANOVA (panel C).

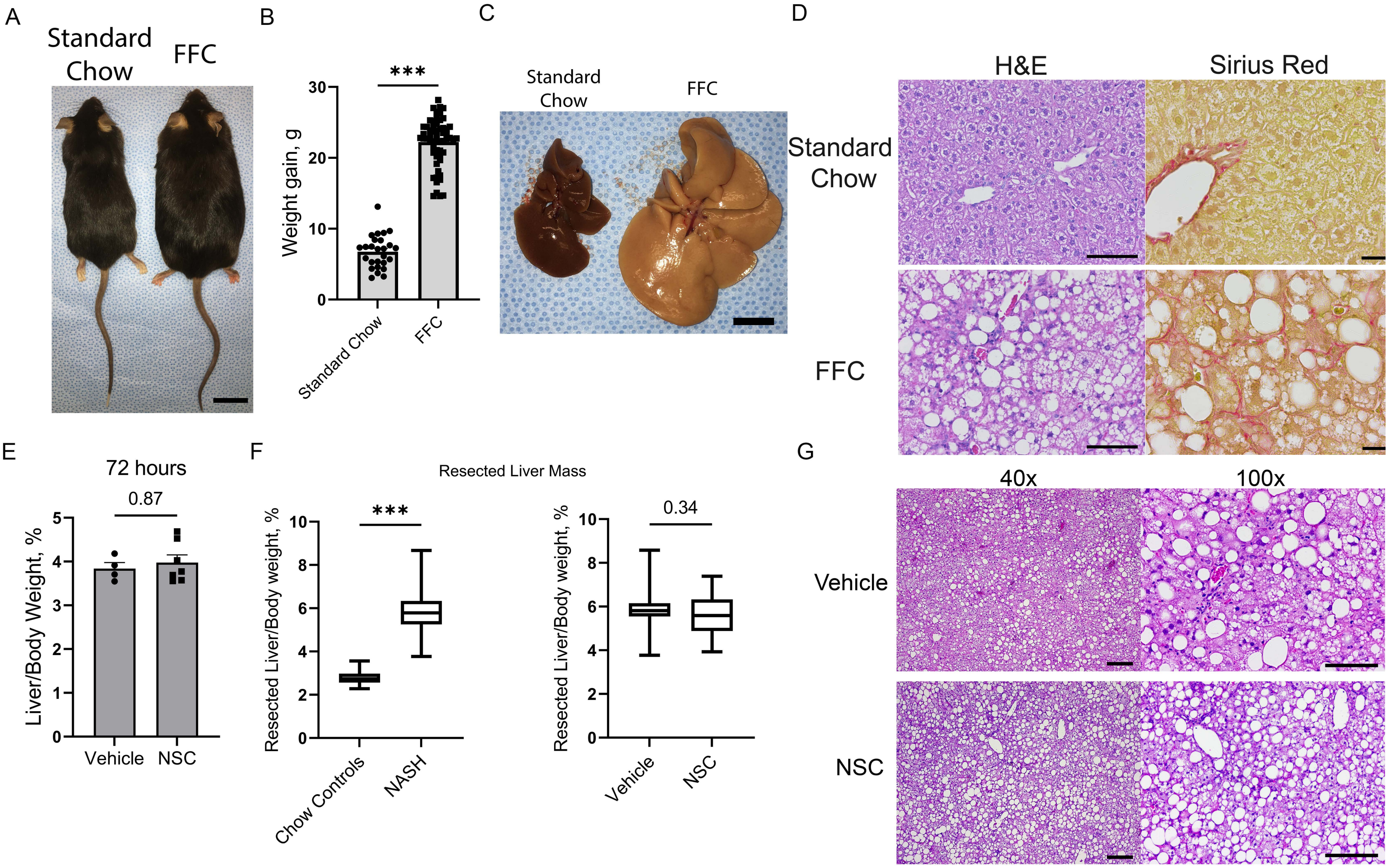

Supplemental Figure 5. Mice display typical characteristics of NASH following FFC diet

A) Representative photograph of C57BL/6 mice fed standard chow or high fat, fructose, and cholesterol diet (FFC) for 24 weeks. Scale bar=2cm. **B)** Average mouse weight gain after 24 weeks on standard chow or FFC diet. **C)** Representative photograph of mouse livers after 24 weeks on standard chow or FFC diet. Scale bar=1cm. **D)** Representative H&E and Sirius red stained liver sections from mice fed standard chow or FFC diet (n=3). Scale bars=50μm **E)** Mean liver to body weight ratio 72-hours post-hepatectomy in NASH mice treated with vehicle (n=4) or NSC (n=8). **F)** Resected liver mass normalized to mouse body weight (median and interquartile range with whiskers at the minimum and maximum value) in all chow control and NASH mice and NASH mice treated with vehicle (n=18) or NSC (n=12). **G)** Representative images at 2 magnifications (40x and 100x) of H&E stain livers from vehicle or NSC treated NASH mice 72-hours post-hepatectomy (n=3). Scale bars=200μm. Graphs represent mean ± SEM (***$p<0.001$). Statistical analysis was performed with student t test. Non-alcoholic steatohepatitis (NASH).

Supplemental Table 2. Antibody list

Antibody	Manufacturer	Cat. #	Application	Dilution
p-YAP (Y357)	Abcam	ab62751	WB	1:1000
p-YAP (S127)	Cell Signaling	4911	WB	1:1000
YAP	Cell Signaling	4912	WB	1:1000
YAP	Santa Cruz	sc-101199	IF	1:500
TAZ	Cell Signaling	4883	WB	1:1000
PCNA	Cell Signaling	13110	WB	1:1000
JNK	Cell Signaling	9252	WB	1:1000
p-JNK (T183/Y185)	Cell Signaling	4671	WB	1:1000
ERK1/2	Cell Signaling	9102	WB	1:1000
p-ERK1/2 (T202/Y204)	Cell Signaling	4370	WB	1:1000
p38	Cell Signaling	8690	WB	1:1000
p-p38 (T180/Y182)	Cell Signaling	4511	WB	1:1000
Src	Cell Signaling	2110	WB	1:1000
p-SRC (Y416)	Cell Signaling	2101	WB	1:1000
p-NR4A1 (S351)	Thermo Sci	PA5-105155	WB	1:1000
NR4A1	Santa Cruz	sc-166166	WB	1:1000
NR4A1	Santa Cruz	sc-166166	IF	1:500
Ki67	Novus Bio	NB110-89717H	IHC	1:50
BrdU	Rockland	600-401-c29	IF	1:200
SHP2	Cell Signaling	3752	IF	1:500
Actin	Santa Cruz	sc-47778	WB	1:1000
GAPDH	Santa Cruz	sc-47724	WB	1:1000
WB=Western blot; IF=Immunofluorescence; IHC=Immunohistochemistry				

Supplemental Table 3. qRT-PCR primer sequences

Primer	Forward (5'-3')	Reverse (5'-3')
**Human**		
CTGF	CAGTGTCTGACTTCGACAACGC	CCATCGGCGTGTTTGGAGTA
CYR61	GAGTGGGTCTGTGACGAGGAT	GGTTGTATAGGATGCGAGGCT
NUAK2	GATGCACATACGGAGGGAGATT	ATCACGATCTTGCTGCTGTTCT
**Mouse**		
Ctgf	CACTCTGCCAGTGGAGTTCA	AAGATGTCATTGTCCCCAGG
Nuak2	CAGGCATTTCTTCCGACAGATC	GAGAGGCCAAAGTCAGCAAT
Nr4a1	TCATCACTGATCGACACG	CTTCAGACAGCTAGCAATG
**Housekeeping**		
18s	CGCTTCCTTACCTGGTTGAT	GAGCGACCAAAGGAACCATA
